# Production of novel beneficial alleles of a rice yield‐related QTL by CRISPR/Cas9

**DOI:** 10.1111/pbi.13370

**Published:** 2020-03-16

**Authors:** Yongtao Cui, Xingming Hu, Guohua Liang, Anhui Feng, Fanmiao Wang, Shuang Ruan, Guojun Dong, Lan Shen, Bin Zhang, Dongdong Chen, Li Zhu, Jiang Hu, Yongjun Lin, Longbiao Guo, Makoto Matsuoka, Qian Qian

**Affiliations:** ^1^ State Key Laboratory of Rice Biology China National Rice Research Institute Hangzhou China; ^2^ National Key Laboratory of Crop Genetic Improvement and National Centre of Plant Gene Research Huazhong Agricultural University Wuhan China; ^3^ Bioscience and Biotechnology Center Nagoya University Nagoya Japan; ^4^ Jiangsu Key Laboratory of Crop Genetics and Physiology/Co‐Innovation Center for Modern Production Technology of Grain Crops Key Laboratory of Plant Functional Genomics of the Ministry of Education Yangzhou University Yangzhou China

**Keywords:** CRISPR‐Cas9, QTL, SCM3/OsTB1/ FC1, Noncoding region

Breeding high‐yield crop cultivars has improved agronomic performance for key grain crops (Hirano *et al.*, 2017). However traditional breeding takes years. Molecular genetic studies have identified beneficial trait‐associated alleles in elite cultivars providing a platform for gene‐editing techniques such as the clustered regularly interspaced short palindromic repeats (CRISPR)/CRISPR‐associated 9 (Cas9) system (Rothan *et al.*, 2019).

Most current ‘super rice’ varieties with high yields have several beneficial agronomic traits. For example the variety Liang‐You‐Pei‐Jiu has strong culms for lodging resistance and large panicles for high yield. Our previous study using recombinant inbred lines (RILs) from a cross between 93‐11 and PA64 identified 43 quantitative trait loci (QTLs) associated with many agronomic traits including heading date spikelet number per panicle and grain shape (Gao *et al.*, 2013). However no QTL for culm strength or panicle size was detected.

Here we studied culm strength by measuring stem cross‐section area (SCSA) of the fourth internode (Figure 1a). By using 127 chromosome substitution segment lines (CSSLs) from a cross between 93‐11 and Nipponbare (NPB) we detected three QTLs associated with SCSA (Figure 1b,c,d) (Xu *et al.*, 2010). The position of *qSCSA1‐1* (Chr1: 42290679‐43345963) overlapped with *STRONG CULM 1* (*SCM1*) (Mulsanti *et al.*, 2018) and *SEMI DWARF‐1* (*SD‐1*) and *qSCSA6‐1* (Chr6: 28410389‐28439277) overlapped with *SCM2* (Figure 1b,d). For both QTLs the alleles from NPB positively contributed to SCSA.

**Figure 1:**
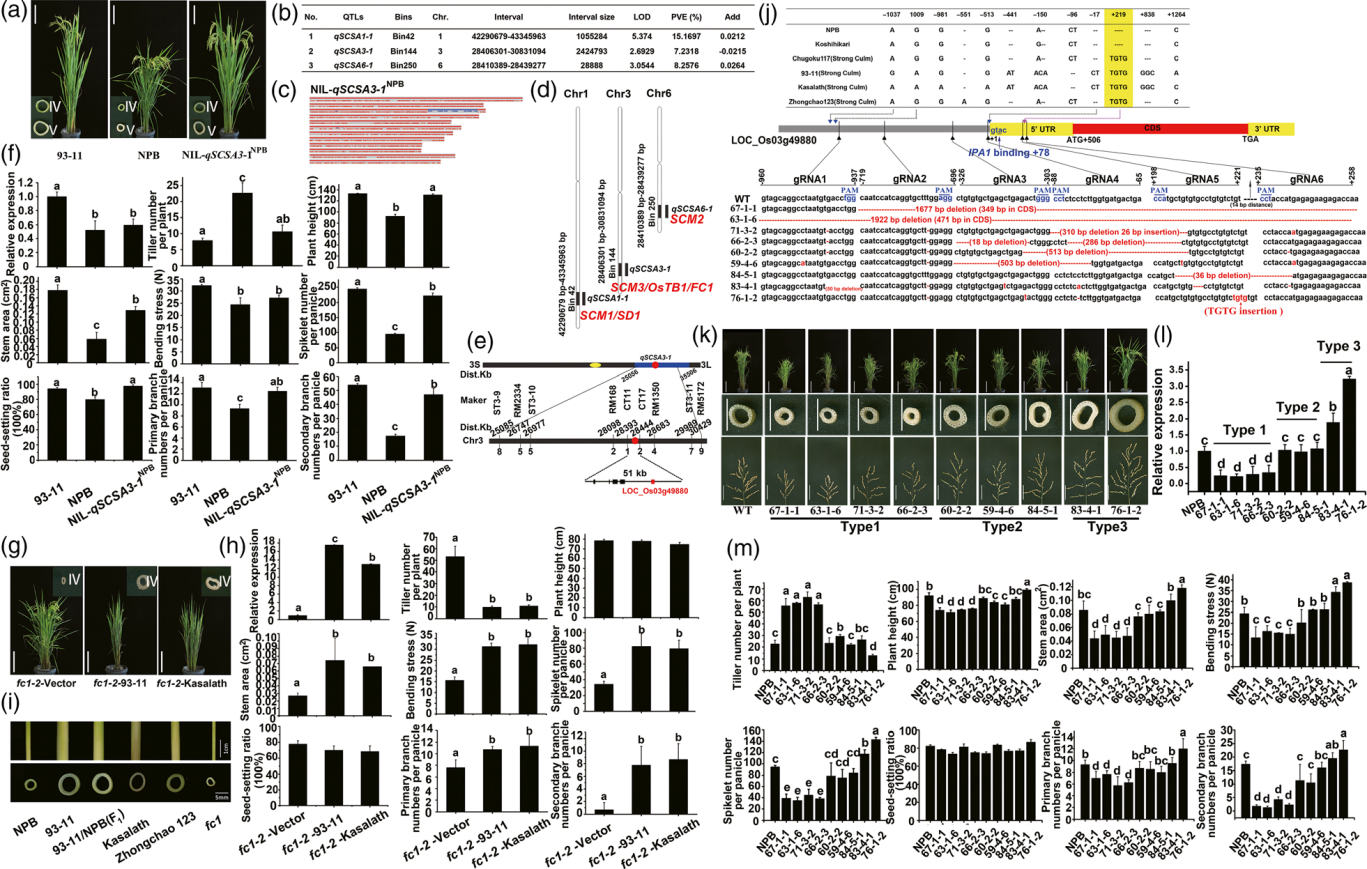
CRISPR‐Cas9 alleles alter *OsTB1* transcript levels and plant phenotypes. (a) Global features and cross‐section of the 4th and 5th internodes (the 2nd and most basal internodes) of 93‐11 (*indica*), NPB (*japonica*) and NIL‐*qSCSA3‐1* ^NPB^. Bar: 20 cm. (b) QTLs for SCSA. (c) NIL‐*qSCSA3‐1* ^NPB^ carrying the NPB allele. (d) Three QTLs associated with SCSA. *qSCSA1‐1, qSCSA3‐1* and *qSCSA6‐1* overlapped with *SCM1/SD1*, *SCM3/OsTB1/FC1* and *SCM2/APO1*, respectively. (e) Mapping of *qSCSA3‐1*. Its candidate region included *OsTB1.* (f) *OsTB1* expression and agronomic traits in 93‐11 (*indica*), NPB (*japonica*), and NIL‐*qSCSA3‐1* ^NPB^. (g) Appearance of plants transformed with the empty vector (left) and its whole genome sequence of 93‐11 (middle) or Kasalath (right). Bar: 20 cm. (h) *OsTB1* expression and agronomic traits in the transgenic plants. (i) The 4th internode phenotype of NPB, 93‐11, 93‐11/NPB(F_1_), Kasalath, Zhongchao 123, *fc1*. Upper panel: 4th internode of NPB, 93‐11, 93‐11/NPB(F_1_), Kasalath, Zhongchao 123, *fc1* (from left to right) Bar = 1 cm. Bottom panel: Cross‐sections of the 4th internode NPB, 93‐11, 93‐11/NPB(F_1_), Kasalath, Zhongchao 123, *fc1* (from left to right) Bar = 5 mm. (j) *OsTB1* genomic region with DNA polymorphisms in four varieties with large SCSA, (9311, Kasalath, Zhongchao123, and Chugoku117), positions of sgRNAs and mutated sequences in the nine genome‐edited plants. *IPA1* target site (GTAC + 78) is indicated. (k) Phenotypes of the edited plants. NPB (leftmost) was used as a control. Top to bottom, plant appearance, cross‐section of the 4th internode and panicle structure. Scale bars, 20 cm, 7 mm and 7 cm, respectively. (l) *OsTB1* expression in edited plants. (m) Agronomic traits in these plants. Error bars represent mean ± SD (n = 3). The different letters indicate statistical differences by Duncan’s multiple range test (p < 0.05). All RNAs were isolated from 2‐cm inflorescences.

The region of *qSCSA3‐1* on the long arm of Chr3 (28406301‐30831094) included *SCM3*/*RICE TEOSINTE BRANCH1* (*OsTB1*)/*FINE CULM1* (*FC1*) (Figure 1b,c,d) (Yano *et al.*, 2015) and the 93‐11 allele had a positive effect on culm strength. To test whether *OsTB1* is the causal gene for *qSCSA3‐1* fine mapped *qSCSA3‐1* using near‐isogenic lines (NILs). *qSCSA3‐1* mapped to a 51‐kb region between markers CT11 and CT17 a region that includes *OsTB1* (Figure 1e). Quantitative‐PCR (qPCR) showed that the expression of *OsTB1* in 93‐11 inflorescences was higher than that of NPB and NIL‐93‐11 carrying the NPB allele NIL‐*qSCSA3‐1^NPB^* (Figure 1f).

In addition to affecting SCSA introgression of the NPB allele resulted in a decrease in bending stress stem area and number of spikelets per panicle and an increase in tiller number but had no effect on plant height (Figure 1f). These observations indicated that *OsTB1* has pleiotropic effects on different traits. Therefore we examined the effect of *OsTB1* using mutant and complemented plants (Figure 1g). The null mutant of *OsTB1* *fc1‐2* showed lower bending stress smaller panicles and increased tiller number compared with rescued plants expressing *OsTB1* from 93‐11 or Kasalath (Figure 1h). These observations confirmed that *qSCSA3‐1* is allelic to *SCM3/OsTB1* and has pleiotropic effects.

We compared the sequence of *OsTB1* among rice cultivars NPB (*japonica*) Koshihikari (*japonica*) and four varieties with large SCSA: 93‐11 (*indica*) Kasalath (*indica*) Zhongchao123 (*japonica*) and Chugoku117 (*indica*) and found polymorphisms in the 5’‐flanking coding sequence (CDS) (Figure 1i,j). Interestingly the four varieties with large SCSA contained a TGTG insertion at + 219 in the 5’‐noncoding region suggesting that this insertion might be important for *OsTB1* expression and consequently affect SCSA.

Based on this prediction we introduced mutations in the promoter and 5’‐noncoding regions of *SCM3/OsTB1* in NPB using CRISPR‐Cas9. We designed six single‐guide RNAs (sgRNAs) four targeting the promoter sequence and two sgRNA5 and 6 targeting the region proximal to the TGTG insertion in the 5’‐flanking CDS. These six sgRNAs were integrated into one plasmid and transformed into NPB using *Agrobacterium*. We obtained 23 plants carrying 9 different mutations (Figure 1j). Relative to wild‐type (WT) NPB Type 1 had more tillers and smaller culms and panicles; Type 2 had similar phenotypes to WT; Type 3 had fewer tillers and larger culms and panicles (Figure 1k). The Type 1 plants 67‐1‐1 63‐1‐6 71‐3‐2 and 66‐2‐3 were similar to the null mutant of *OsTB1* *fc1‐2* (Minakuchi *et al.*, 2010). Indeed 71‐3‐2 and 66‐2‐3 contained deletions in the 5’‐noncoding region of *OsTB1*. OsSPL14/IPA1 positively regulates *OsTB1* expression through binding to a GTAC motif (Lu *et al.*, 2013) and this motif was deleted in these plants likely resulting in the null phenotypes.

The Type 3 plants 83‐4‐1 and 76‐1‐2 mimicked the phenotypes of NIL*^SCM3^* (Yano *et al.*, 2015) and 93‐11 suggesting that mutations in these plants enhance *OsTB1* expression. Indeed these plants showed higher *OsTB1* expression than the control whereas the Type 1 and Type 2 plants showed reduced and unchanged expression levels respectively (Figure 1l). 76‐1‐2 had a TGTG insertion which also occurs in varieties with large SCSA (Figure 1i j) confirming that the TGTG insertion enhances *OsTB1* expression. Although 83‐4‐1 had mutations at six sites in the 5’‐flanking CDS region it showed a gain‐of‐function phenotype (Figure 1k). Thus different sequence(s) in the 5’‐flanking CDS region in WT may repress or enhance transcription. In 84‐5‐1 a 36‐bp deletion around the TGTG insertion did not increase *OsTB1* expression suggesting that the TGTG insertion did not disrupt a repressive site. Instead the TGTG insertion may create a binding site for transcription activator(s) hypotheses that will require further validation.

For Type 2 plants which showed similar phenotypes to the control plants (Figure 1k) *OsTB1* expression did not differ from the control although they had various deletions and single‐nucleotide polymorphisms upstream of the gene (Figure 1j). These results demonstrated that targeted editing of *OsTB1 cis*‐regulatory elements could produce alleles having different expression levels and phenotypes (Figure 1l m).

The application of CRISPR‐Cas9 for studying *cis*‐elements has been discussed but a detailed strategy has not been established in rice (Rodriguez‐Leal *et al.*, 2017). In this study we introduced mutations in *cis*‐regulatory sequences which led to alterations in gene expression and phenotypes and reproduced beneficial alleles in new rice varieties. QTL studies have identified beneficial alleles in rice elite germ plasm including alleles affecting gene expression. For example the molecular mechanism for major QTLs controlling plant structure such as *GN1A IPA1/WFP NUMBER OF GRAINS 1* (*NOG1*) *FZP1/qSRN7* and also *SCM2/APO1* as mentioned above depends on the difference in the expression level of these genes (Huo *et al.*, 2017). These spontaneous alleles have been introgressed into elite varieties by breeding which is time‐consuming and labour‐intensive. CRISPR‐Cas9 however easily produced alleles with similar expression levels as shown for *OsTB1.* Furthermore we may be able to isolate desirable alleles with more appropriate levels of expression for optimizing breeding targets. In this regard saturation editing of known genes associated with agronomic traits of interest may accelerate molecular design of desirable traits.

## Authors’ contributions

Xingming Hu, Qian Qian and Makoto Matsuoka conceived the project and designed the research strategies. Yongtao Cui, Xingming Hu, Lan Shen, Fanmiao Wang, Shuang Ruan and Anhui Feng cloned the CRISPR‐Cas9 plasmid, identified mutants and performed qRT‐PCR. Guohuang Liang, Bin Zhang, Yongjun Lin, Dongdong Chen, Li Zhu and Longbiao Guo performed the SASA QTL analysis and mapping. Qian Qian, Xingming Hu, Guojun Dong and Jiang Hu performed phenotype observations and genetic tests. Xingming Hu, Makoto Matsuoka and Qian Qian wrote the article.

## Competing interests

The authors declare no competing interests.

## Supporting information


**Figure S1** Mapping of SCSA (Stem Cross‐Section Area) QTLs CSSLs.
**Figure S2** Identification of hygromycin in different editedplants.
**Table S1** Primer sequences used in this study.
**Data S1** Materials and Methods.
