# Supplementary material for: Production of novel beneficial alleles of a rice yield‐related QTL by CRISPR/Cas9
Source: Plant Biotechnol J. 2020 Mar 16;18(10):1987–9. doi: 10.1111/pbi.13370 (PMC7540660; doi:10.1111/pbi.13370)
Supplement: Supplementary file 1 — Figure S1 Mapping of SCSA (Stem Cross‐Section Area) QTLs CSSLs. Figure S2 Identification of hygromycin in different editedplants. Table S1 Primer sequences used in this study. Data S1 Materials and Methods. [file PBI-18-1987-s001.pdf]

Supplementary information

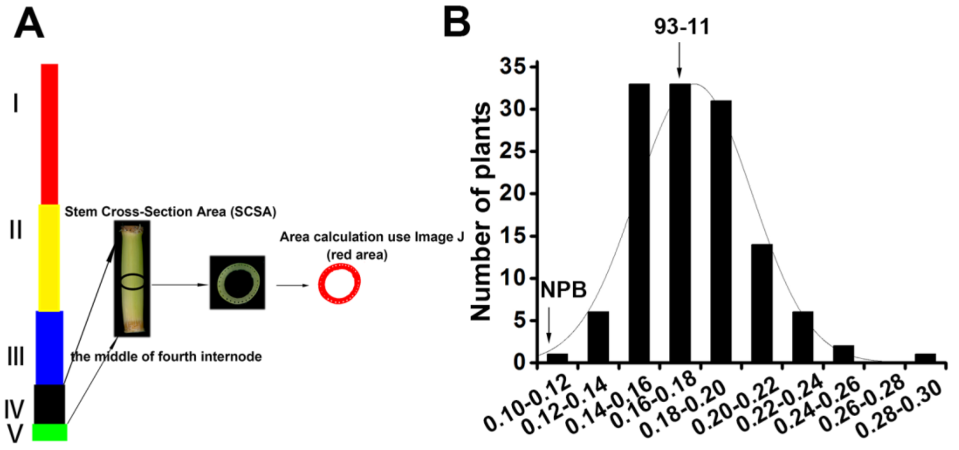

Figure S1. Mapping of SCSA (Stem Cross-Section Area) QTLs CSSLs.

(A): Measurement of SCSA. Schematic illustration of the middle of 4th internodes (IN) were sectioned at the basal position and the section modulus area was measured by digital image processing software (ImageJ64 version 1.45). (B): Frequency distribution of SCSA of the 4th internode of 127 CSSLs.

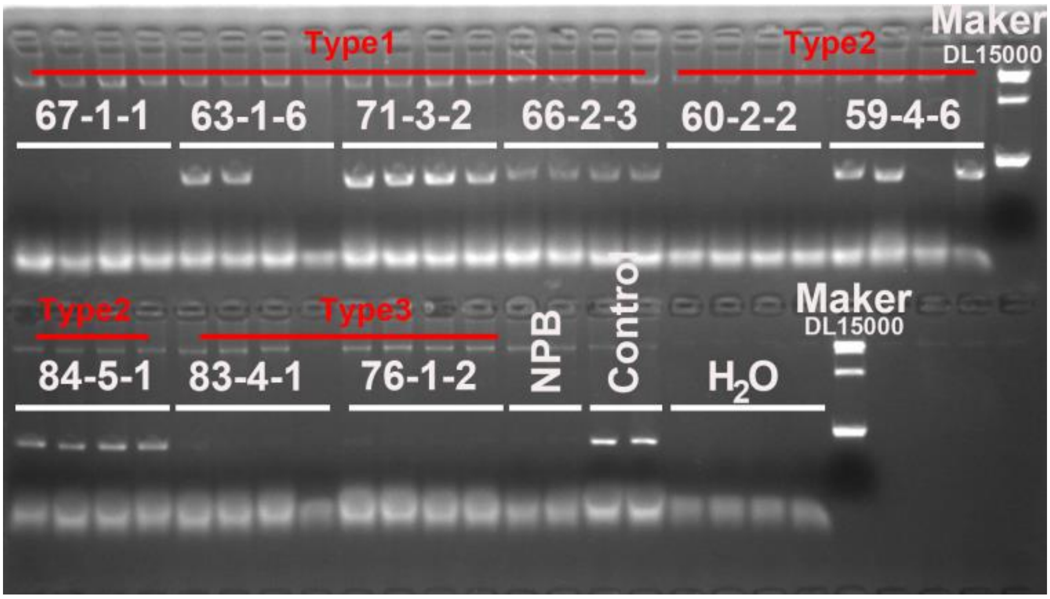

Figure S2: Identification of hygromycin in different edited plants. Type 1, 2, and 3, based on the phenotypes (see text).

Table S1 Primer sequences used in this study.

Primer name	Primer sequence (5'-3')
S3-9F	AGCCAACACATAAGGAGAAAAC
S3-9R	GTAAGGTTGACCATCAGCACTAT
RM2334F	TGTGAAGAGTACAAGTAGGG
RM2334R	CATGCATCTGATCTGATTAT
S3-10F	TAAGCCAGGTCCCATCAA
S3-10R	GACCAACTACAACACCAATACAA
RM168F	TGCTGCTTGCCTGCTTCCTTT
RM168R	GAAACGAATCAATCCACGGC
CT11F	TGACATAGTGAAAAATTTTAGGG
CT11R	GTAAATATTTCACGAGATTCCAA
CT17F	TGCTAAGTCTCTCCTTCGTATCAC
CT17R	CCAACTGATATTGCGAATAG
RM1350F	AGGAAATTCGCCCTAGTAGATAG
RM1350R	ATCAGCAAGAAAGCTCTGCTCC
S3-11F	AGTTTTCGCAGCAGTTAGGGT
S3-11R	CATCTCTCCCATCGTGCGTA
RM5172F	TGGCTGTTATGTGAAATACAC
RM5172R	CTCTGTGATGTATTAGGGAAAAC
OsTB1 complementation F	ccatgattacgaattcATATTATAGGAAATAACATATATTGAAGTC
OsTB1 complementation R	gcctgcaggtcgactctagaACAGTACTTTCTTATTGGTGCATT
OsTB1 4bp check F	TCCTAATCCTCCTACCCACC
OsTB1 4bp check R	CTTGGTTGATGCTTGGTCTC
qPCR-OsTB1F	TCATCCATCCACACACGAAC
qPCR-OsTB1R	ATGCGATGACCAAACCAAAG
OsUBQ-F	AACCAGCTGAGGCCCAAGA
OsUBQ-R	ACGATTGATTTAACCAGTCCATGA
OsTB1-H1-gRNA	GTAGCAGGCCTAATGTGACCTGG
OsTB1-H2-gRNA	CAATCCATCAGGTGCTTTGGAGG
OsTB1-H3-gRNA	CTGTGTGCTGAGCTGAGACTGGG
OsTB1-H4-gRNA	TCAGTCATCACCAAGAGGAGAGG
OsTB1-H5-gRNA	ACAGACACAGGCACACAGCATGG
OsTB1-H6-gRNA	TTGGTCTCTTCTCTCATGGTAGG
OsTB1 -site-idF	GAGATGGCGCCGATACCTTAT
OsTB1 -site-idR	GGGACTCCTTACTCCTAGCC

Materials and Methods

Plant materials and growth conditions

A *japonica* variety (Nipponbare) and an *indica* variety with strong culms (93-11) were used in this study. The *fc1-2* (defective in strigolactone signaling) mutant was provided by Dr. Junko Kyozuka. The Chromosome Segment Substitution Line (CSSL) populations were grown at the experimental farm of China National Rice Research Institute in Hangzhou (119°54′ E, 30°04′ N) and Lingshui (110°00′ E, 18°31′ N).

Histological Analysis

Sections were observed and photographed with ScanWizard EZ (ScanMaker i800; MICROTEK), and area was measured using digital image processing software (ImageJ64 version 1.45). The bending stress was measured using a pulling tool (DS2-200N; IMADA).

DNA Extraction and Molecular Analysis

Genomic DNA was extracted from leaves of each individual using the cetyltrimethylammoniumbromide (CTAB) method (Rogers and Bendich, 1989). The DNA amplification was performed by PCR as follows: pre-denaturation at 94°C for 4 min; 40 cycles of denaturation at 94°C for 30 s, annealing at 55–60°C for 30 s and extension at 72°C for 30 s, with a final extension at 72°C for 10 min. The reactions were carried out in 96-well PCR plates in 10-μl volumes containing 50–100 ng template DNA, 0.2 μmol/l of each primer and 5 μl of 2× Taq Master Mix (Vazyme Inc.). Electrophoresis of the amplification products was carried out on 4% agarose gels and photographed using GelDoc XR Gel Documentation System (Bio-Rad Inc.). The markers used were InDel markers developed from a BLASTN alignment between the genome sequence of three parents. Primers were designed with Primer3 (http://primer3.ut.ee/).

QTL Analysis for Stem Cross-Section Area Based on CSSLs

Bin maps were converted from the physical maps of CSSLs for QTL analysis. Most donor segments between or among the different lines of each CSSL have a little overlap. To perform QTL analysis, the overlapping chromosome segments of the CSSLs were used to delineate smaller segment sizes that were described as bins (Huang et al., 2009). The QTL analysis was performed from these bins, which served as genetic markers, using QTL IciMapping V4.0 (www.isbreeding.net/software/). The likelihood ratio test based on stepwise regression for the additive QTL (RSTEP-LRT-ADD) method was employed for power analysis. The mapping parameters of probability in stepwise regression was set at 0.001, and the multicollinearity control by condition number was set at 1000 as the default. The LOD threshold for each dataset was based on a permutation test (1000 permutations, $P = 0.05$). When the QTL LOD score was larger than 2.5, the QTL was designated as having a major effect.

Fine mapping of *qSCSA3-1*

Based on the primary QTLs detected, we crossed the CSSL (C015) with 93-11. Seventeen BC_5F_2 plants carrying chromosomal recombination events between ST3-9 and RM5172 showed low SCSA measured by ImageJ64 version 1.45. The plants showing low SCSA were backcrossed with 93-11. Twelve BC_6F_2 recombinant plants carrying chromosomal recombination events between ST3-10 and ST3-11 showed low SCSA, *qSCSA3-1* was localized to a 300-kb interval between ST3-10 and ST3-11. Self-pollination of BC_6F_2 plants, which were heterozygous between the markers ST3-10 and ST3-11. We fine mapped *qSCSA3-1* between the markers RM168 and RM1350 by using BC_6F_3 and BC_6F_4. After using a final set of 9 recombinant BC_6F_5 plants segregated from BC_6F_4 that were heterozygous between the markers RM168 and RM1350, the candidate region was localized to 50 kb between CT11 and CT17, where five candidate genes were found. Molecular markers for genotyping are listed in Table S1.

RNA extraction and RT-PCR

Total RNA was extracted from 2-cm long young panicle tissues using TRIzol reagent (Thermo Fisher Scientific). The first-strand cDNA was synthesized using 1 μg RNA and ReverTra Ace qPCR RT Master Mix with gDNA Remover (Toyobo), according to the manufacturer's instructions. Real-time PCR was carried out for each cDNA replicate and all samples were run in duplicate. The quantitative real-time PCR experiments were performed using a Power SYBR Green PCR Master Mix kit (Thermo Fisher Scientific) and the following conditions: 95°C for 10 min; followed by 40 cycles of 95°C for 15 s and 60°C for 1 min; with a final melt curve generated between 60°C and 95°C. The rice *UBIQUITIN* gene was used as an internal control. The relative expression ratios were obtained using the equation $2^{-\Delta\Delta CT}$. The gene-specific primers used are shown in Table S1.

Vector construction and plant transformation

All PCR primers used for plasmid construction are listed in Table S1. The 2.5-kb DNA fragment upstream of *OsTB1* amplified from 93-11 or Kasalath and the 1.3-kb 3′ genomic sequence were amplified from 93-11 or Kasalath and cloned into the binary vector pCAMBIA1300 to generate the p*93-11*::*OsTB1* expression cassette or p*Kasalath*::*OsTB1* expression cassette.

For the construction of the plasmids expressing sgRNAs-Cas9 variants, six guide RNAs were designed to target the *OsTB1* promoter region. The rice-transforming plasmid system was previously described (Shen et al., 2017). The targeted sequences are listed in Table S1. Transgenic rice plants were generated using Agrobacterium-mediated transformation (Hiei et al., 1997).

Detection of mutations

Genomic DNA of transgenic plants was extracted from approximately 100 mg leaf tissue of rice via the CTAB method. PCR was conducted with KOD FX DNA polymerase

(Toyobo, Japan) to amplify the fragments surrounding the target sites. The DNA fragments were sequenced by the Sanger method and analyzed by the degenerate sequence decoding method (Liu et al., 2015).

Hiei, Y., Komari, T. and Kubo, T. (1997) Transformation of rice mediated by Agrobacterium tumefaciens. *Plant molecular biology* **35**, 205-218.

Huang, X., Feng, Q., Qian, Q., Zhao, Q., Wang, L., Wang, A., Guan, J., Fan, D., Weng, Q. and Huang, T. (2009) High-throughput genotyping by whole-genome resequencing. *Genome research* **19**, 1068.

Liu, W., Xie, X., Ma, X., Li, J., Chen, J. and Liu, Y.G. (2015) DSDecode: A Web-based Tool for Decoding of Sequencing Chromatograms for Genotyping of Targeted Mutations. *Molecular plant* **8**, 1431-1433.

Rogers, S.O. and Bendich, A.J. (1989) *Extraction of DNA from plant tissues*.

Shen, L., Hua, Y., Fu, Y., Li, J., Liu, Q., Jiao, X., Xin, G., Wang, J., Wang, X., Yan, C. and Wang, K. (2017) Rapid generation of genetic diversity by multiplex CRISPR/Cas9 genome editing in rice. *Science China Life sciences* **60**, 506-515.
